# Risk factors and comorbidities associated with magnesium deficiency in pregnant women and women with hormone-related conditions: analysis of a large real-world dataset

**DOI:** 10.1186/s12884-021-03558-2

**Published:** 2021-01-22

**Authors:** Svetlana Orlova, Galina Dikke, Gisele Pickering, Natalya Yaltseva, Sofya Konchits, Kirill Starostin, Alina Bevz

**Affiliations:** 1 Department of Dietetics and Clinical Nutritiology of Continuing Medical Education, Medical Institute, RUDN University, Moscow, Russia; 2 Department of Obstetrics and Gynecology with a course of Reproductive Medicine, The Academy of Medical Education. F.I. Inozemtsev, Saint-Petersburg, Russia; 3 Department of Clinical Pharmacology Inserm CIC 1405, University Hospital, Clermont-Ferrand, France; 4 Department of General Medicine, The Yaroslavl State Medical University Institute of Postgraduate Education, Yaroslavl, Russia; 5 Department of Medical Affairs, Sanofi, Moscow, Russia

**Keywords:** Magnesium deficiency, Osteoporosis, Climacteric syndrome, Pregnancy, Hormone replacement therapy, Oral contraceptives

## Abstract

**Background:**

An accumulating body of literature indicates that magnesium deficiency is associated with a number of hormone-related conditions (HRC) in women, and epidemiological studies are needed to assess its prevalence and risk factors. Here, we present a secondary analysis of data pooled from four large observational studies that assessed magnesium deficiency among pregnant women and women with HRC across the Russian Federation.

**Methods:**

The main objective of this analysis was to estimate the prevalence of magnesium deficiency in this population and to describe risk factors and comorbidities associated with low serum magnesium. Univariate logistic regression analysis was performed to identify the risk factors and comorbid conditions associated with an increased risk of low serum magnesium level.

**Results:**

A total of 983 pregnant women and 9444 women with HRC were eligible for analysis. Prevalence of hypomagnesemia (magnesium serum level cut-off < 0.66 mmol/L/< 0.8 mmol/L) was 34.0%/78.9% in pregnant women and 21.4%/54.8% in women with HRC. The highest prevalence of magnesium deficiency was observed for osteoporosis and climacteric syndrome. Risk factors included diastolic blood pressure, previous pregnancy complications, infections and edema for pregnant women, and age, body mass index, and various comorbidities for women with HRC.

**Conclusions:**

These results confirm the high prevalence of hypomagnesemia in pregnant women and women with HRC and underline the importance of routine screening, since risk factors are mostly non-specific.

**Supplementary Information:**

The online version contains supplementary material available at 10.1186/s12884-021-03558-2.

## Background

Magnesium is one of the essential minerals and is involved in a plethora of metabolic functions. Magnesium deficiency is associated with a range of diseases and conditions, such as hypertension, diabetes, neurological and cardiovascular event [1]. It is estimated that 48–60% of adults do not achieve the average recommended dietary intake of magnesium, and 15–42% of apparently healthy individuals have been shown to have magnesium deficiency [2–4]. Magnesium deficiency is more frequent in women than men [2, 4]; this may partially be influenced by the fact that estrogen stimulates magnesium utilization by tissues and therefore hormonal rhythms in women may affect and modulate magnesium status [5].

Assessing magnesium levels may present a challenge because of its predominant retention in soft tissues and bones. Magnesium in the blood only accounts for about 0.8% of all magnesium in the body, with 0.3% contained in serum and 0.5% in red blood cells [4]. Since magnesium level is most frequently assessed by measuring blood serum concentration, magnesium deficiency may be masked by apparently normal serum levels and its prevalence may be underestimated [1, 3, 6]. Furthermore, there is no uniform lower reference limit for serum magnesium level, with cut-offs ranging between 0.66 mmol/L and 0.85 mmol/L in different studies [2, 7, 8].

Although the majority of adults will not experience serious consequences, subclinical magnesium deficiency may have a more profound effect on individuals in high risk groups, e.g. pregnant women and women undergoing menopause, who are at risk of osteoporosis [2]. Pregnancy is associated with an increased magnesium requirement resulting from a combination of fetal demand, altered tissue distribution and an increased renal output of magnesium [9]. It is recommended to closely monitor magnesium levels in pregnant women with kidney disease [10]. Recent studies suggest that low magnesium during pregnancy may be associated with adverse maternal and fetal outcomes including preeclampsia and fetal growth retardation, although there is no consensus on the benefit of magnesium supplementation in preventing these outcomes [9, 11–15]. An accumulating body of literature indicates that magnesium deficiency may also be linked to various other aspects of women’s health, such as menopause, osteoporosis and use of oral contraceptives [16, 17].

Although the importance of magnesium in pregnancy and various hormone-related conditions is widely recognized, further studies are needed to establish the prevalence of magnesium deficiency and to what extent low magnesium levels can be linked to patient outcomes and comorbidities. To this end, four large observational studies were conducted between 2012 and 2016 across the Russian Federation. The studies assessed the prevalence and clinical management of magnesium deficiency in pregnant women (MAGIC, MAGIC2) and in women with hormone-related conditions (MAGYN, MAGYN2) using a magnesium deficiency questionnaire (MDQ) and a blood test assessing serum magnesium levels [18–21].

These studies showed an unexpectedly high prevalence of magnesium deficiency, assessed using both MDQ and blood magnesium test [18–21]. Here, we present the results of a secondary analysis of the prevalence of magnesium deficiency in women with hormone-related conditions and pregnant women with symptoms of magnesium deficiency in the pooled population and evaluate its association with risk factors and comorbidities related to magnesium deficiency in these cohorts.

## Methods

### Study design and patients

This manuscript summarizes a part of the secondary analysis of pooled data collected in four observational studies of magnesium deficiency in pregnant women and women with hormone-related conditions: MAGIC (DIREGL06157), MAGIC2 (DIREGL06468), MAGYN (MAGNEL06863), and MAGYN2 (MAGNEL07741) [18–21].

MAGIC and MAGIC2 enrolled pregnant women (*N* = 1130 and *N* = 2117, respectively) during routine visits to maternity welfare centers. Women were included in the studies if they were > 18 years of age, were pregnant and had suspected magnesium deficiency (fatigue, muscle cramps, etc.). The study excluded women who reported other known or obvious reasons for magnesium deficiency beside pregnancy [19, 21]. MAGYN and MAGYN2 studies enrolled women with hormone-related conditions (*N* = 9168 and *N* = 11,424, respectively) attending outpatient clinics. Women were included if they were 18–60 years of age and used hormonal contraception or hormone replacement therapy (HRT) or had one of the following conditions: premenstrual syndrome (PMS), climacteric syndrome without HRT, osteoporosis or other hormonal conditions (including endometriosis, polycystic ovarian disease, uterine leiomyoma, dysmenorrhea, endometrial hyperplasia). Women were excluded if they had severe conditions potentially hindering participation in the study or were receiving magnesium supplementation at baseline [18, 20].

**Fig. 1:**
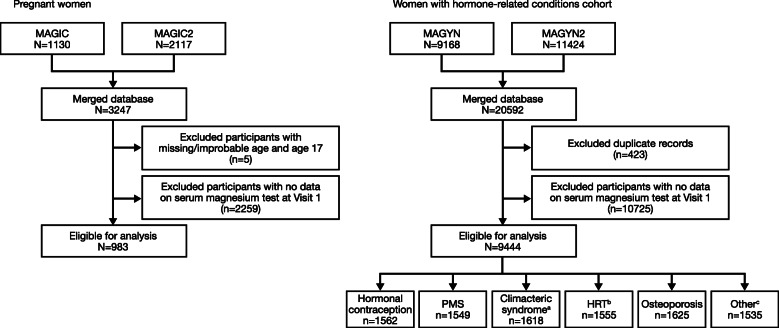
Study cohorts. ^a^Includes women with climacteric syndrome not receiving HRT. ^b^Includes surgical menopause. ^c^Includes women of reproductive age with other hormonal conditions: endometriosis, polycystic ovarian disease, uterine leiomyoma, dysmenorrhea, endometrial hyperplasia. HRT, hormone replacement therapy; PMS, premenstrual syndrome

The present analysis included all patients who fulfilled the inclusion/exclusion criteria in the studies (Fig. 1). Patients with missing data, contradictory/inconsistent data or outlier data were excluded from the analysis (exclusion was performed separately for each variable of interest). Patient characteristics, available medical history and serum magnesium test results were combined in two pooled databases (‘pregnant women’ and ‘women with hormone-related conditions’). Medical history that was available for the two cohorts of participants included any recorded terms that were relevant for this group: for example, for the ‘pregnant women’ cohort, the terms included complications and complaints associated with a previous pregnancy, whereas for the cohort of ‘women with hormone-related conditions’ the terms included general diseases and comorbidities. The rate of magnesium deficiency was estimated using two cut-offs: 0.66 mmol/L, the traditional lower reference limit used in the Russian Federation and other countries [7], and 0.8 mmol/L, the lower reference limit that has been recommended based on the recent studies [3, 8].

### Study objectives

The objectives of the secondary analysis presented here were to characterize magnesium status, estimate the prevalence of hypomagnesemia and identify possible hypomagnesemia risk factors and associated comorbidities in pregnant women and in women with hormone-related conditions.

### Statistical analysis

Study cohorts were characterized using descriptive statistics. The cohort of pregnant women was treated as a single entity, whereas the cohort of women with hormone-related conditions was separated into subgroups according to their specific conditions. The prevalence of hypomagnesemia was assessed in both cohorts and in the subgroups of women with hormone-related conditions. All subgroups of women with hormone-related conditions were crosschecked for conflicting data on concomitant diseases, laboratory and clinical assessments; patients with missing, conflicting or improbable data were excluded from analysis.

Differences in magnesium levels between cohorts and subgroups were analyzed using the chi square test, unpaired t-test and non-parametric tests. Univariate logistic regression analysis was performed to identify the risk factors and comorbid conditions associated with an increased risk of low serum magnesium level within both cohorts. The results were expressed as odds ratios with 95% confidence intervals. Statistical significance threshold was set at *p* < 0.05; the Bonferroni correction was applied to correct for the numerous multiple testing, leading to thresholds of *p* < 0.0005 for pregnant women (up to 100 tests) and *p* < 0.0001 for women with hormone-related conditions (up to 500 tests).

## Results

### Study cohorts

In total, 983 participants in the ‘pregnant women’ cohort and 9444 participants in the ‘women with hormone-related conditions’ cohort were eligible for analysis. Women with hormone-related conditions included the following subgroups: women receiving hormonal contraception (*n* = 1562; among them, only 74 women were taking progestin-only formulations), women with premenstrual syndrome (*n* = 1549), women with climacteric syndrome not receiving HRT (*n* = 1618), women receiving HRT, including surgical menopause (*n* = 1555), women with osteoporosis (*n* = 1625) and women with other hormone-related conditions, such as endometriosis, polycystic ovarian disease, uterine leiomyoma, dysmenorrhea, endometrial hyperplasia (*n* = 1535) (Fig. 1).

Participants in the ‘pregnant women’ cohort had median age 28.0 (min–max 18–52), median body mass index (BMI) 23.1 kg/m^2^ (min–max 15.9–50.3 kg/m^2^) and mean (standard deviation [SD]) serum magnesium level 0.714 (0.125) mmol/L (Table 1).

**Table 1:** Baseline characteristics and magnesium status of study participants

	Pregnant women (***N*** = 983)	Women with hormone-related conditions						
		Hormonal contraception (***n*** = 1562)	Premenstrual syndrome (***n*** = 1549)	Climacteric syndrome^**a,b**^ (***n*** = 1618)	HRT^**b**^ (***n*** = 1555)	Osteoporosis (***n*** = 1625)	Other^**c**^ (***n*** = 1535)	Total (***N*** = 9444)
**Age, years**								
n/missing	983/0	1562/0	1549/0	1618/0	1555/0	1625/0	1535/0	9444/0
Median	28.0	30.0	29.0	51.	50.0	55.0	36.0	44.0
Q1; Q3	25.0; 32.0	25.0; 34.0	25.0; 35.0	48.0; 54.0	47.0; 54.0	51.0; 58.0	30.0; 42.0	31.0; 52.0
Min; Max	18; 52	18; 55	18; 56	29; 60	23; 60	19; 60	18; 59	18; 60
**BMI, kg/m**^**2**^								
n/missing	983/0	1554/8	1540/9	1609/9	1546/9	1620/5	1532/3	9401/43
Median	23.10	22.10	22.40	27.20	26.30	27.10	24.20	25.00
Q1; Q3	20.90; 26.40	20.30; 24.60	20.30; 25.20	24.70; 30.50	24.00; 29.00	24.30; 30.50	21.80; 27.40	22.00; 28.30
Min; Max	15.9; 50.3	14.6; 48.5	15.6; 43.7	15.2; 63.4	13.8; 60.6	15.2; 51.8	14.8; 52.1	13.8; 63.4
**Total blood serum magnesium, mmol/L**								
n/missing	983/0	1562/0	1549/0	1618/0	1555/0	1625/0	1535/0	9444/0
Mean (SD)	0.714 (0.125)	0.789 (0.197)	0.787 (0.193)	0.765 (0.198)	0.787 (0.219)	0.758 (0.199)	0.771 (0.180)	0.776 (0.198)
Median	0.700	0.780	0.790	0.750	0. 780	0.750	0.770	0.770
Q1; Q3	0.650; 0.780	0.680; 0.900	0.680; 0.890	0.650; 0.860	0.680; 0.890	0.640; 0.860	0.660; 0.890	0.660; 0.890
Min; Max	0.12; 1.92	0.09; 2.41	0.14; 2.50	0.20; 2.50	0.10; 4.08	0.20; 2.20	0.08; 2.50	0.08; 4.08

*BMI* Body mass index, *HRT* Hormone replacement therapy, *Q* Quartile

^a^Women with climacteric syndrome not receiving HRT

^b^Including surgical menopause

^c^Women of reproductive age with other hormonal conditions: endometriosis, polycystic ovarian disease, uterine leiomyoma, dysmenorrhea, endometrial hyperplasia

Participants in the ‘women with hormone-related conditions’ cohort had median age 44.0 years (min–max 18–60 years), median BMI 25.0 kg/m^2^ (min–max 13.8–63.4 kg/m^2^) and mean (SD) serum magnesium level 0.776 (0.198) mmol/L (Table 1). Among different subgroups, women with osteoporosis, women with climacteric syndrome and women receiving HRT were on average older and had higher BMI than women in the other subgroups.

### Magnesium levels and prevalence of magnesium deficiency

Participants in the ‘pregnant women’ cohort had lower mean serum magnesium levels than ‘women with hormone-related conditions’ (0.714 mmol/L [SD = 0.125 mmol/L] vs 0.776 mmol/L [SD = 0.198 mmol/L], *p* < 0.0001). In the ‘women with hormone-related conditions’ cohort, the highest mean total serum magnesium level was found in the subgroup of women receiving hormonal contraception (0.789 mmol/L [SD = 0.197 mmol/L]), and the lowest – in women with osteoporosis (0.758 mmol/L [SD = 0.199 mmol/L]) (Table 1). The differences across subgroups were statistically significant (*p* < 0.0001).

**Fig. 2:**
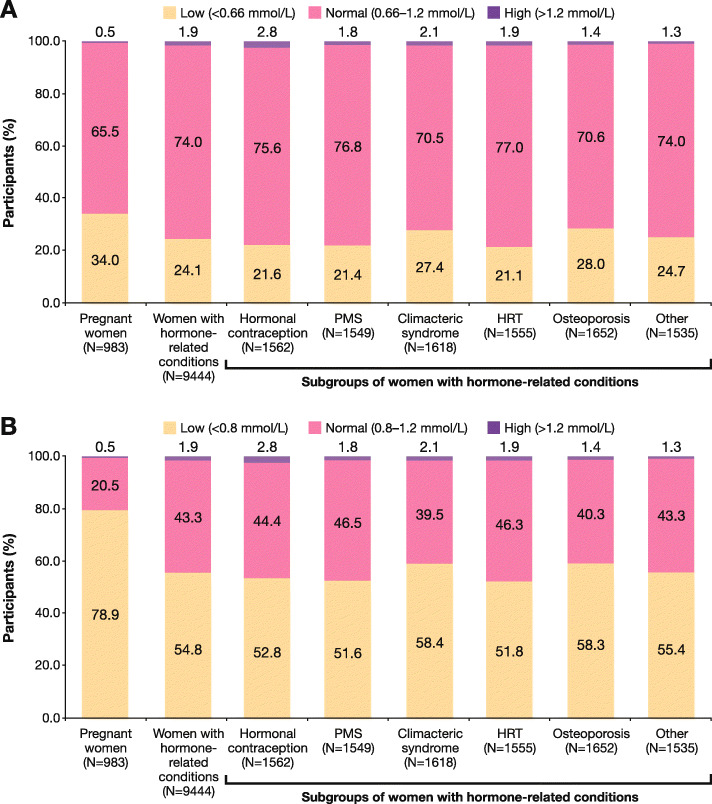
Proportion of participants according to serum magnesium status at baseline using 0.66 mmol/L **a** or 0.8 mmol/L **b** as the cut-off. HRT, hormone replacement therapy; PMS, premenstrual syndrome

Prevalence of magnesium deficiency assessed by serum levels in ‘pregnant women’ cohort was 34.0% or 78.9% when using 0.66 mmol/L or 0.8 mmol/L as the cut-off, respectively (Fig. 2). Prevalence of magnesium deficiency assessed by serum blood levels in ‘women with hormone-related conditions’ cohort was 24.1% or 54.8% when using 0.66 mmol/L or 0.8 mmol/L as the cut-off, respectively (Fig. 2). Among all subgroups, the highest prevalence of magnesium deficiency was observed among women with osteoporosis (28.0% using cut-off < 0.66 mmol/L and 58.3% using cut-off < 0.8 mmol/L) and women with climacteric syndrome (27.4 and 58.4%, respectively).

### Potential risk factors associated with low serum magnesium levels in pregnant women

**Fig. 3:**
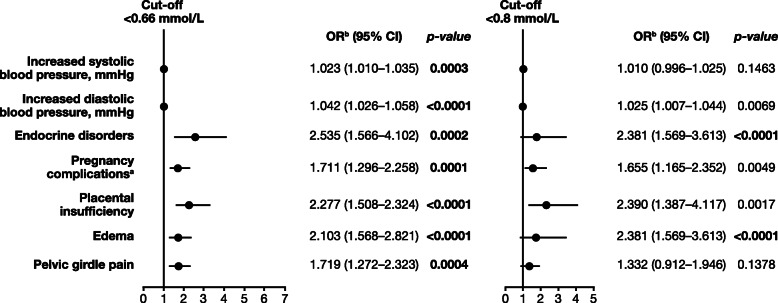
Risk factors significantly associated with hypomagnesemia defined by a cut-off of < 0.66 mmol/L in pregnant women. ^a^In the past medical history. ^b^Estimated using logistic regression. CI, confidence interval; OR, odds ratio

In the ‘pregnant women’ cohort, several risk factors and comorbidities showed a statistically significant association with low serum magnesium levels (*p* ≤ 0.0005; Supplementary Table 1). For the cut-off of < 0.66 mmol/L, significant associations included increase in systolic blood pressure (*p* = 0.0003), increase in diastolic blood pressure (*p* < 0.0001), endocrine disorders (*p* = 0.0002), previous pregnancy complications (such as placental insufficiency [*p* < 0.0001]), and complaints (edema [*p* < 0.0001] and pelvic girdle pain [*p* = 0.0004]). Edema was the only risk factor significantly associated with hypomagnesemia defined by the cut-off of < 0.8 mmol/L (*p* < 0.0001) (Fig. 3**;** Supplementary Table 1).

### Potential risk factors associated with low serum magnesium levels in women with hormone-related conditions

In the ‘women with hormone-related conditions’ cohort, a large number of risk factors and comorbidities showed a statistically significant association with low serum magnesium (26 factors for cut-off < 0.66 mmol/L and 38 factors for cut-off < 0.8 mmol/L; Supplementary Table 2). Factors associated with both cut-offs included age and BMI (*p* < 0.0001 for all). All studied symptoms of magnesium deficiency were significantly associated with low serum magnesium (*p* < 0.0001 for all). Furthermore, the potential risk factors included various comorbidities and previous obstetric and gynecological conditions outlined below.

The association was significant for the following comorbidities: gastrointestinal diseases (hepatitis [*p* < 0.0001 for both cut-offs] and cholelithiasis [*p* < 0.0001 for both cut-offs]), urolithiasis [*p* < 0.0001 for both cut-offs], cardiovascular diseases (pathology of heart valves [*p* ≤ 0.0001 for both cut-offs]), and hypothyroidism (*p* < 0.0001 for both cut-offs) (Supplementary Table 2).

**Fig. 4:**
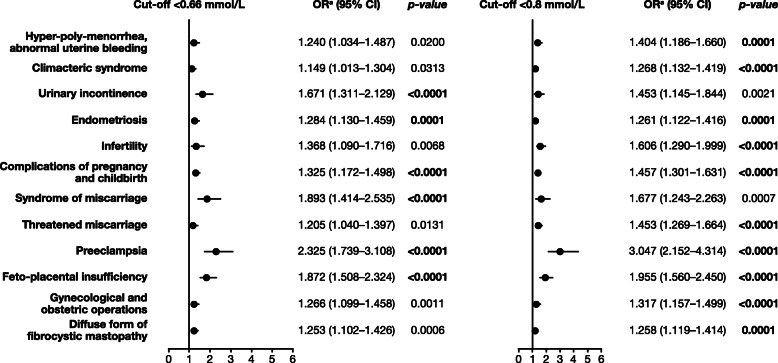
Risk factors from the category ‘obstetric and gynecological past medical history and status’ associated with hypomagnesemia defined by a cut-off of < 0.66 mmol/L in women with hormone-related conditions. CI, confidence interval; OR, odds ratio. ^a^Estimated using logistic regression

The association was also significant for previous obstetric and gynecological conditions, such as endometriosis (*p* = 0.0001 for both cut-offs) and complications of pregnancy and childbirth (including preeclampsia [*p* < 0.0001 for both cut-offs] and feto-placental insufficiency [*p* < 0.0001 for both cut-offs]), (Fig. 4).

Among factors that defined the subgroups of ‘women with hormone-related conditions’, osteoporosis was significantly associated with hypomagnesemia using the cut-off of 0.66 mmol/L (*p* < 0.0001). Associations for all risk factors and both cut-offs are listed in the Supplementary Table 2.

### Magnesium supplementation

Participants of observational studies MAGIC, MAGIC2, MAGYN and MAGYN2 received magnesium supplements prescribed by treating physicians in accordance to routine clinical practice; a proportion of participants received magnesium-vitamin B6 combination (Magne B_6_/Magne B_6_ Forte) [18–21]. The effectiveness results are presented in detail elsewhere [22]. Briefly, after taking magnesium supplements for 4 weeks, 293 of 318 (92.1%) pregnant women with initial level < 0.66 mmol/L achieved magnesium level ≥ 0.66 mmol/L, and 529 of 717 (73.8%) pregnant women with initial level < 0.8 mmol/L achieved magnesium level ≥ 0.8 mmol/L. The corresponding proportions among women with hormone-related conditions were 78.4% (283 or 361 women) and 58.9% (452 of 767 women), respectively.

## Discussion

This study was one of the largest and the most comprehensive real-world studies of magnesium deficiency in women. The study cohorts consisted of pregnant women and women with hormone-related conditions from multiple cities and regions of the Russian Federation, providing wide geographical coverage and a large sample size (a total of 10,427 women).

The prevalence of magnesium deficiency in the studied population was extremely high, reaching 78.9% in pregnant women and 54.8% in women with hormone-related conditions. These rates are among the highest cited in literature; however, one should keep in mind that participants of MAGIC and MAGIC2 studies were enrolled based on clinical suspicion of magnesium deficiency. Furthermore, the analysis of MAGYN and MAGYN2 study included only women with serum magnesium level assessment at Visit 1, i.e. those with suspected magnesium deficiency. Nevertheless, these results complement other studies, including those assessing magnesium levels in general adult populations of Germany, Mexico, Taiwan and the USA that found higher prevalence of hypomagnesemia in women (21.0–40.0%) than in men (1.5–35.4%) [2, 23–25]. In these studies, magnesium deficiency was determined using cut-offs between 0.76 mmol/L and 0.85 mmol/L [2, 23–25]. High rates of hypomagnesemia have been reported in pregnant women in India (43.6%) and Sudan (57.2%); both studies used a cut-off of 0.74 mmol/L [26, 27]. The results of the present study confirm the general observations that pregnant women, women receiving oral contraceptives, postmenopausal women and women with osteoporosis are at a particular risk of hypomagnesemia [9, 16, 17]. The multifactorial reasons for the increased risk during pregnancy have been discussed in detail elsewhere and include fetal demand, altered tissue distribution and an increased renal output of magnesium [9]. High estrogen levels may also influence magnesium utilization in tissues and may account for subnormal serum magnesium levels in young women receiving oral contraceptives, with multiple gender-specific physiological ageing processes accounting for low magnesium in women with osteoporosis [4, 17]. Irrespective of the underlying cause, there is an argument for the use of magnesium supplementation in women who are at risk of hypomagnesemia, including pregnant women and women with hormone-related conditions. Notably, among various subgroups of women with hormone-related conditions, women with osteoporosis had the highest incidence of hypomagnesemia (28.0%/58.3% using cut-off < 0.66/0.8 mmol/L), but also had the highest rate of achieving target serum magnesium level with magnesium supplementation (88.1%/58.8% using cut-off < 0.66/0.8 mmol/L). These results emphasize a possible role for magnesium supplementation in the prevention or treatment of various gynecological conditions that could be explored in future studies.

Several risk factors associated with low magnesium levels have been identified in the studied cohorts. The broad variety of conditions and comorbidities found in this study are in line with the previous studies in pregnant women and the general population (e.g. cardiovascular comorbidities) and further expand the current knowledge of the effect of hypomagnesemia on women’s health. In pregnant women, risk factors for both 0.66 mmol/L and 0.8 mmol/L cut-offs included increased diastolic blood pressure, previous pregnancy complications, such as preeclampsia and placental insufficiency, and edema. Previous studies have identified placental insufficiency, preeclampsia, miscarriage, premature birth and gestational diabetes as conditions and outcomes associated with low magnesium levels [9]. Magnesium sulphate is recommended by the WHO for the prevention and treatment of eclampsia [28]. However, despite its wide use in clinical practice, the dosing regimens vary across countries and are often inconsistent with the international recommendations [29]. Our results further strengthen the clinical evidence supporting a direct link between magnesium supplementation and risk of preeclampsia and may help facilitate the uptake of the WHO guidelines throughout the world. It may also be interesting to test in further clinical studies whether the use of over-the-counter magnesium supplements in pregnant women decreases the risk of preeclampsia and the need for intravenous magnesium administration.

In women with hormone-related conditions, risk factors for both 0.66 mmol/L and 0.8 mmol/L cut-offs included age and BMI, all studied symptoms of magnesium deficiency, and a number of previous gynecological conditions and general comorbidities. Our analysis found significant associations between the risk of low magnesium and various general comorbidities, including general stress (various laboratory and hormone values) in women with hormone-related conditions. These observations support previous reports linking immune dysfunction and general stress to magnesium deficiency [30, 31]. The large number of risk factors associated with hypomagnesemia in this study confirms the status of magnesium as one of the essential elements in health and supports further clinical research investigating the role of magnesium in various neurological, gastrointestinal and cardiovascular conditions.

This study has several limitations. The observational studies MAGIC and MAGIC2 collected data on pregnant women with suspected magnesium deficiency, and it is not possible to generalize these results to the overall population. However, the study generated an important insight into hypomagnesemia in a larger cohort of women with hormone-related conditions (MAGYN and MAGYN2). Because of the retrospective observational design of this study, no causal links can be established between magnesium deficiency and associated conditions. Due to the retrospective nature of the analysis, only data that were recorded previously were included, and it was not possible to acquire new information on patient medical history. For instance, viral infections and edema were included in medical history in general terms, and no details were reported with regard to specific viruses or the edema site. These results should therefore be interpreted with caution.

Because the study included participants with suspected magnesium deficiency, the risk of selection bias was high, and the actual prevalence of hypomagnesemia in Russia may be lower than revealed in this study. However, similarly high prevalence of hypomagnesemia has been reported previously [26, 27]. Additionally, pregnancy and hormone-related conditions could be considered as risk factors for magnesium deficiency in general. One may argue that the use of a high cut-off of < 0.8 mmol/L resulted in an artificially increased prevalence detected in this study. The appropriate cut-off has been a matter of debate in literature [3, 8], and future studies will undoubtedly contribute to a consensus on the most clinically relevant cut-off value. Another limitation of this work is that the analysis did not take into account environmental risk factors and social aspects which may have contributed to magnesium deficiency in the study population. Finally, univariate analysis was selected as the simplest method that could provide an exploratory descriptive assessment of potential risk factors for hypomagnesemia. The choice of univariate analysis may be considered a limitation; further studies should include a confirmatory multivariate analysis of potential risk factors.

## Conclusions

This is one of the largest and most representative analyses of magnesium deficiency in pregnant women and women with hormone-related conditions. The study provided an estimate of the prevalence of hypomagnesemia in these cohorts and identified multiple risk factors and associated comorbidities, providing unique insights into the epidemiology of magnesium deficiency in the Russian Federation. These results call for further studies of the prevalence and epidemiology of hypomagnesemia among the general population.

## Supplementary Information

**Additional file 1.**

## Data Availability

Qualified researchers may request access to patient-level data and related documents. Data may be shared upon request by contacting corresponding author (Kirill Starostin). Patient-level data are anonymized, and study documents will be redacted to protect the privacy of trial participants. Further details on Sanofi’s data sharing criteria, eligible studies, and process for requesting access can be found at https://www.clinicalstudydatarequest.com. Other part of this study (Orlova et al, 2020) dedicated to magnesium deficiency questionnaire analysis and shortening is published recently and the reader may be kindly referred to that manuscript as well (see reference #22).

## Abbreviations

BMI: Body mass index
CI: Confidence interval
MDQ: Magnesium deficiency questionnaire
HRC: Hormone-related conditions
HRT: Hormone replacement therapy
OR: Odds ratio
PMS: Premenstrual syndrome
Q: Quartile
SD: Standard deviation
WHO: World Health Organization
