# Supplementary material for: Risk factors and comorbidities associated with magnesium deficiency in pregnant women and women with hormone-related conditions: analysis of a large real-world dataset
Source: BMC Pregnancy Childbirth. 2021 Jan 22;21:76. doi: 10.1186/s12884-021-03558-2 (PMC7821493; doi:10.1186/s12884-021-03558-2)
Supplement: Supplementary file 1 — Additional file 1. [file 12884_2021_3558_MOESM1_ESM.docx]

**Svetlana Orlova,^1^ Galina Dikke,^2^ Gisele Pickering,^3,4^ Natalya Yaltseva,^5^ Sofya Konchits,^6^ Kirill Starostin,^6^ Alina Bevz^6^**

# Risk factors and comorbidities associated with magnesium deficiency in pregnant women and women with hormone-related conditions: Analysis of a large real-world dataset

# Supplementary information

**Supplementary Table 1.** Associations between risk factors and hypomagnesemia in pregnant women.

|  |  | **Cut-off <0.66 mmol/L** | | | **Cut-off <0.8 mmol/L** | | |
| --- | --- | --- | --- | --- | --- | --- | --- |
|  | **N** | **OR^a^** | **95% CI** | ***p-value*** | **OR^a^** | **95% CI** | ***p-value*** |
| Increased systolic blood pressure, mmHg | 981 | 1.023 | 1.010–1.035 | **0.0003** | 1.01 | 0.996–1.025 | 0.1463 |
| Increased diastolic blood pressure, mmHg | 981 | 1.042 | 1.026–1.058 | **<0.0001** | 1.025 | 1.007–1.044 | 0.0069 |
| Increased heart rate | 981 | 0.987 | 0.969–1.005 | 0.1666 | 1.023 | 1.001–1.045 | 0.0405 |
| Dysmenorrhea, Yes vs No | 982 | 1.246 | 0.892–1.740 | 0.1968 | 2.288 | 1.411–3.711 | 0.0008 |
| STI, Yes vs No | 982 | 1.304 | 0.978–1.741 | 0.0709 | 1.492 | 1.039–2.143 | 0.0302 |
| Uterine fibroids, Yes vs No | 982 | 1.740 | 1.116–2.713 | 0.0145 | 1.196 | 0.679–2.105 | 0.5353 |
| Endocrine disorders, Yes vs No | 982 | 2.535 | 1.566–4.102 | **0.0002** | 1.732 | 0.872–3.438 | 0.1164 |
| Abnormal uterine bleeding, Yes vs No | 982 | 4.969 | 1.547–15.966 | 0.0071 | 3.488 | 0.454–26.781 | 0.2297 |
| Other, Yes vs No | 982 | 0.488 | 0.272–0.875 | 0.0161 | 0.721 | 0.417–1.248 | 0.2429 |
| Pregnancy complications^b^, Yes vs No | 982 | 1.711 | 1.296–2.258 | **0.0001** | 1.655 | 1.165–2.352 | 0.0049 |
| Preeclampsia, Yes vs No | 982 | 1.644 | 1.109–2.439 | 0.0134 | 2.743 | 1.444–5.210 | 0.0020 |
| Bleeding postpartum period, Yes vs No | 982 | 2.723 | 1.085–6.835 | 0.0329 | n/a | n/a | n/a |
| Placental insufficiency, Yes vs No | 982 | 2.277 | 1.595–3.250 | **<0.0001** | 2.390 | 1.387–4.117 | 0.0017 |
| Intrauterine growth retardation, Yes vs No | 982 | 3.016 | 1.435–6.339 | 0.0036 | 7.952 | 1.078–58.680 | 0.0420 |
| Hypothyroidism, Yes vs No | 982 | 1.669 | 1.057–2.636 | 0.0278 | 1.603 | 0.851–3.018 | 0.1438 |
| Hepatitis, Yes vs No | 982 | 0.936 | 0.498–1.760 | 0.8385 | 1.503 | 0.662–3.412 | 0.3295 |
| Asthenic syndrome, Yes vs No | 982 | 1.645 | 1.176–2.301 | 0.0037 | 1.448 | 0.935–2.243 | 0.0973 |
| Viral infections, Yes vs No | 982 | 1.461 | 1.099–1.942 | 0.0090 | 1.434 | 1.006–2.046 | 0.0464 |
| Complaints |  |  |  |  |  |  |  |
| Edema, Yes vs No | 982 | 2.103 | 1.568–2.821 | **<0.0001** | 2.381 | 1.569–3.613 | **<0.0001** |
| Pelvic girdle pain, Yes vs No | 982 | 1.719 | 1.272–2.323 | **0.0004** | 1.332 | 0.912–1.946 | 0.1378 |

N, number of patients included in the analysis.

^a^Estimated using logistic regression. ^b^In the past medical history.

ALT, alanine aminotransferase; AST, aspartate aminotransferase; BMI, body mass index; CI, confidence interval; OR, odds ratio; STI, sexually transmitted infection.

**Supplementary Table 2.** Associations between risk factors and hypomagnesemia in women with hormone-related conditions.

|  |  | **Cut-off <0.66 mmol/L** | | | **Cut-off <0.8 mmol/L** | | |
| --- | --- | --- | --- | --- | --- | --- | --- |
|  | **N** | **OR^a^** | **95% CI** | ***p-value*** | **OR^a^** | **95% CI** | ***p-value*** |
| Age, years | 9371 | 1.012 | 1.008–1.016 | **<0.0001** | 1.010 | 1.006–1.013 | **<0.0001** |
| BMI, kg/m^2^ | 9328 | 1.028 | 1.018–1.038 | **<0.0001** | 1.021 | 1.012–1.030 | **<0.0001** |
| Hormonal contraception^b^, Yes vs No | 9371 | 0.846 | 0.742–0.964 | 0.0124 | 0.905 | 0.812–1.010 | 0.0738 |
| Hormonal contraception^b^ adjusted for age and BMI, Yes vs No | 9328 | 1.027 | 0.886–1.190 | 0.7274 | 1.059 | 0.937–1.198 | 0.3579 |
| Premenstrual syndrome^b^, Yes vs No | 9371 | 0.924 | 0.820–1.041 | 0.1950 | 0.859 | 0.770–0.958 | 0.0064 |
| Premenstrual syndrome^b^ adjusted for age and BMI, Yes vs No | 9328 | 1.13 | 0.986–1.295 | 0.0791 | 1.099 | 0.979–1.233 | 0.1084 |
| Climacteric syndrome without HRT^c^, Yes vs No | 9371 | 1.234 | 1.093–1.394 | 0.0007 | 1.195 | 1.072–1.333 | 0.0013 |
| HRT^c^, Yes vs No | 9371 | 0.899 | 0.797–1.014 | 0.0842 | 0.871 | 0.780–0.972 | 0.0133 |
| HRT^c^ adjusted for age and BMI, Yes vs No | 9328 | 0.805 | 0.709–0.913 | 0.0007 | 0.866 | 0.778–0.965 | 0.0093 |
| Osteoporosis^b^, Yes vs No | 9371 | 1.382 | 1.233–1.549 | **<0.0001** | 1.200 | 1.076–1.338 | 0.0010 |
| Other hormonal conditions^d^, Yes vs No | 9371 | 1.034 | 0.911–1.175 | 0.6013 | 1.022 | 0.915–1.141 | 0.7027 |
| Multivitamin supplements, Yes vs No | 9371 | 1.212 | 1.075–1.366 | 0.0017 | 1.549 | 1.389–1.728 | **<0.0001** |
| Oral Combined Contraceptives, type 2-phase vs 1-phase | 1836 | 0.863 | 0.626–1.189 | 0.3667 | 1.173 | 0.902–1.527 | 0.2341 |
| Oral Combined Contraceptives, type 3-phase vs 1-phase | 1836 | 0.871 | 0.525–1.446 | 0.5937 | 1.181 | 0.778–1.792 | 0.4353 |
| **Magnesium deficiency symptoms, Yes vs No** | | | | | | | |
| Weakness | 7371 | 1.117 | 1.092–1.141 | **<0.0001** | 1.177 | 1.153–1.202 | **<0.0001** |
| Sleep disturbance | 6655 | 1.130 | 1.107–1.154 | **<0.0001** | 1.138 | 1.116–1.160 | **<0.0001** |
| Tinnitus | 3654 | 1.231 | 1.188–1.276 | **<0.0001** | 1.261 | 1.215–1.310 | **<0.0001** |
| Tachycardia | 4614 | 1.162 | 1.132–1.193 | **<0.0001** | 1.165 | 1.136–1.194 | **<0.0001** |
| Hot flashes | 4524 | 1.099 | 1.074–1.123 | **<0.0001** | 1.103 | 1.082–1.126 | **<0.0001** |
| Excessive sweating | 5408 | 1.081 | 1.057–1.105 | **<0.0001** | 1.102 | 1.081–1.125 | **<0.0001** |
| Suffocation | 3104 | 1.189 | 1.144–1.236 | **<0.0001** | 1.201 | 1.155–1.249 | **<0.0001** |
| Numbness of limbs | 5145 | 1.157 | 1.125–1.190 | **<0.0001** | 1.238 | 1.204–1.273 | **<0.0001** |
| Irritability | 7934 | 1.060 | 1.040–1.081 | **<0.0001** | 1.090 | 1.071–1.109 | **<0.0001** |
| Hair loss, brittle nails | 6428 | 1.058 | 1.033–1.083 | **<0.0001** | 1.086 | 1.064–1.109 | **<0.0001** |
| Convulsions of lower limbs | 5332 | 1.154 | 1.126–1.183 | **<0.0001** | 1.227 | 1.197–1.257 | **<0.0001** |
| Sleep disorders | 6572 | 1.123 | 1.100–1.147 | **<0.0001** | 1.125 | 1.104–1.147 | **<0.0001** |
| Chronic stress | 6113 | 1.089 | 1.068–1.111 | **<0.0001** | 1.149 | 1.128–1.170 | **<0.0001** |
| Muscle weakness | 5879 | 1.132 | 1.106–1.159 | **<0.0001** | 1.215 | 1.187–1.243 | **<0.0001** |
| Feeling a lump in the throat | 3175 | 1.197 | 1.150–1.247 | **<0.0001** | 1.284 | 1.229–1.341 | **<0.0001** |
| Stomach ache, abdominal cramps | 4255 | 1.069 | 1.039–1.101 | **<0.0001** | 1.094 | 1.067–1.122 | **<0.0001** |
| Back pain | 5551 | 1.122 | 1.096–1.148 | **<0.0001** | 1.137 | 1.113–1.161 | **<0.0001** |
| Dizziness | 5059 | 1.135 | 1.104–1.167 | **<0.0001** | 1.193 | 1.161–1.226 | **<0.0001** |
| Paresthesia | 4007 | 1.246 | 1.205–1.288 | **<0.0001** | 1.338 | 1.291–1.386 | **<0.0001** |
| Fast fatiguability | 7422 | 1.081 | 1.059–1.103 | **<0.0001** | 1.131 | 1.110–1.152 | **<0.0001** |
| Tremor | 3072 | 1.314 | 1.257–1.373 | **<0.0001** | 1.427 | 1.354–1.504 | **<0.0001** |
| Frequent headaches | 5695 | 1.106 | 1.082–1.129 | **<0.0001** | 1.145 | 1.122–1.168 | **<0.0001** |
| Chvostek's sign | 2289 | 1.559 | 1.402–1.734 | **<0.0001** | 2.959 | 2.339–3.744 | **<0.0001** |
| **Clinical and laboratory tests performed within 28–30 days prior to enrolment (data from medical records)** | | | | | | | |
| Abnormalities on ultrasound of the pelvis, Yes vs No | 8196 | 0.911 | 0.821–1.012 | 0.0809 | 1.128 | 1.030–1.234 | 0.0091 |
| Abnormalities on ECG, Yes vs No | 5493 | 1.563 | 1.334–1.832 | **<0.0001** | 1.637 | 1.401–1.912 | **<0.0001** |
| Hemoglobin, g/L | 8295 | 0.990 | 0.985–0.994 | **<0.0001** | 0.988 | 0.984–0.992 | **<0.0001** |
| Platelets, 10^9^/L | 7815 | 1.003 | 1.003–1.004 | **<0.0001** | 1.002 | 1.001–1.003 | **<0.0001** |
| Erythrocyte sedimentation rate, mm/h | 8127 | 1.018 | 1.008–1.028 | 0.0003 | 1.033 | 1.024–1.043 | **<0.0001** |
| ALT, U/L | 6476 | 1.005 | 1.000–1.009 | 0.0381 | 1.005 | 1.000–1.009 | 0.0392 |
| AST, U/L | 6399 | 1.008 | 1.003–1.013 | 0.0008 | 1.009 | 1.004–1.014 | 0.0007 |
| Total protein, g/L | 6701 | 0.996 | 0.988–1.004 | 0.3144 | 0.994 | 0.987–1.001 | 0.0831 |
| Glucose, mmol/L | 7720 | 1.245 | 1.172–1.322 | **<0.0001** | 1.259 | 1.188–1.335 | **<0.0001** |
| Total bilirubin, μmol/L | 6596 | 1.013 | 1.006–1.020 | 0.0002 | 1.003 | 0.996–1.010 | 0.3710 |
| Total cholesterol, mmol/L | 6322 | 1.168 | 1.113–1.225 | **<0.0001** | 1.225 | 1.171–1.280 | **<0.0001** |
| Osteocalcin, ng/mL | 1725 | 1.018 | 1.011–1.024 | **<0.0001** | 1.033 | 1.025–1.041 | **<0.0001** |
| FSH, mU/L | 4004 | 1.000 | 0.998–1.002 | 0.7963 | 1.001 | 0.999–1.003 | 0.4728 |
| LH, mU/L | 3524 | 1.002 | 1.000–1.005 | 0.0753 | 1.002 | 0.999–1.006 | 0.1200 |
| Estradiol, pg/mL | 3121 | 1.001 | 1.000–1.002 | 0.0621 | 1.001 | 1.000–1.001 | 0.1824 |
| Progesterone, nmol/L | 2599 | 0.998 | 0.995–1.002 | 0.4231 | 0.997 | 0.993–1.000 | 0.0601 |
| Testosterone, nmol/L | 2701 | 1.000 | 0.988–1.011 | 0.9553 | 0.982 | 0.972–0.993 | 0.0009 |
| SHBG, nmol/L | 1486 | 0.999 | 0.996–1.003 | 0.7022 | 0.996 | 0.992–0.999 | 0.0067 |
| Calcitonin, pg/mL | 1534 | 1.024 | 1.011–1.037 | 0.0004 | 1.055 | 1.036–1.073 | **<0.0001** |
| Parathormone, pg/mL | 1385 | 1.005 | 1.000–1.010 | 0.0542 | 0.998 | 0.993–1.003 | 0.4044 |
| **Comorbidities (general history), Yes vs No** | | | | | | | |
| Frequent viral infections | 9371 | 1.097 | 0.970–1.241 | 0.1405 | 1.190 | 1.067–1.326 | 0.0017 |
| Allergic reactions | 9371 | 1.096 | 0.945–1.271 | 0.2234 | 1.292 | 1.132–1.475 | **0.0001** |
| Bronchial asthma | 9371 | 1.662 | 1.276–2.163 | 0.0002 | 1.884 | 1.437–2.470 | **<0.0001** |
| Scoliosis, flat feet | 9371 | 1.053 | 0.881–1.258 | 0.5704 | 1.090 | 0.933–1.274 | 0.2793 |
| Myopia | 9371 | 1.214 | 1.078–1.368 | 0.0014 | 1.219 | 1.095–1.357 | 0.0003 |
| ENT diseases | 9371 | 1.000 | 0.872–1.146 | 1.000 | 1.033 | 0.919–1.163 | 0.5840 |
| Anemia | 9371 | 1.359 | 1.130–1.636 | 0.0012 | 1.654 | 1.384–1.976 | **<0.0001** |
| Type 1 diabetes | 9371 | 2.282 | 1.196–4.351 | 0.0123 | 2.615 | 1.237–5.528 | 0.0118 |
| Type 2 diabetes | 9371 | 1.424 | 0.994–2.040 | 0.0538 | 1.312 | 0.932–1.847 | 0.1195 |
| Dyslipidemia | 9371 | 1.559 | 1.177–2.064 | 0.0019 | 1.925 | 1.444–2.564 | **<0.0001** |
| Obesity | 9371 | 1.290 | 1.107–1.504 | 0.0011 | 1.392 | 1.208–1.604 | **<0.0001** |
| Varicose veins | 9371 | 1.184 | 1.031–1.359 | 0.0169 | 1.168 | 1.032–1.323 | 0.0139 |
| Thrombosis | 9371 | 1.491 | 0.874–2.544 | 0.1431 | 1.202 | 0.722–2.000 | 0.4788 |
| Vegetative-vascular dystonia | 9371 | 1.090 | 0.965–1.231 | 0.1659 | 1.120 | 1.006–1.246 | 0.0379 |
| Asthenic syndrome | 9371 | 0.966 | 0.762–1.225 | 0.7778 | 1.216 | 0.990–1.494 | 0.0623 |
| Gastrointestinal diseases | 9371 | 1.301 | 1.175–1.440 | **<0.0001** | 1.356 | 1.238–1.485 | **<0.0001** |
| Hepatitis | 9371 | 2.501 | 1.756–3.561 | **<0.0001** | 2.904 | 1.905–4.427 | **<0.0001** |
| Cholelithiasis | 9371 | 1.633 | 1.371–1.946 | **<0.0001** | 1.443 | 1.217–1.712 | **<0.0001** |
| Other diseases of the liver and bile ducts | 9371 | 1.320 | 1.040–1.675 | 0.0224 | 1.720 | 1.365–2.168 | **<0.0001** |
| Chronic gastritis, chronic gastroduodenitis | 9371 | 1.051 | 0.934–1.182 | 0.4123 | 1.199 | 1.081–1.329 | 0.0006 |
| Gastric and duodenal ulcer | 9371 | 1.268 | 0.943–1.705 | 0.1167 | 1.143 | 0.871–1.500 | 0.3342 |
| Other | 9371 | 0.432 | 0.169–1.103 | 0.0792 | 1.408 | 0.745–2.662 | 0.2922 |
| Kidneys and urinary tract diseases | 9371 | 1.209 | 1.079–1.355 | 0.0011 | 1.359 | 1.227–1.506 | **<0.0001** |
| Chronic pyelonephritis | 9371 | 1.174 | 1.013–1.361 | 0.0333 | 1.318 | 1.153–1.507 | **0.0001** |
| Urolithiasis | 9371 | 1.628 | 1.317–2.013 | **<0.0001** | 1.722 | 1.392–2.130 | **<0.0001** |
| Cystitis | 9371 | 0.935 | 0.780–1.120 | 0.4662 | 1.112 | 0.953–1.298 | 0.1770 |
| Other | 9371 | 1.171 | 0.310–4.416 | 0.8161 | 3.655 | 0.789–16.923 | 0.0975 |
| Neurological diseases | 9371 | 1.271 | 1.141–1.415 | **<0.0001** | 1.199 | 1.089–1.319 | 0.0002 |
| Osteochondrosis | 9371 | 1.269 | 1.140–1.414 | **<0.0001** | 1.194 | 1.085–1.315 | 0.0003 |
| Stroke | 9371 | 1.646 | 0.764–3.545 | 0.2028 | 1.000 | 0.480–2.081 | 1.000 |
| Other | 9371 | 0.224 | 0.030–1.695 | 0.1472 | 0.927 | 0.336–2.558 | 0.8831 |
| Cardiovascular diseases | 9371 | 1.376 | 1.214–1.559 | **<0.0001** | 1.378 | 1.228–1.547 | **<0.0001** |
| Atherosclerosis | 9371 | 2.113 | 1.594–2.800 | **<0.0001** | 1.547 | 1.159–2.064 | 0.0031 |
| Arterial hypertension, hypertonic disease | 9371 | 1.263 | 1.098–1.454 | 0.0011 | 1.263 | 1.111–1.435 | 0.0003 |
| Heart rhythm disturbance | 9371 | 1.501 | 1.166–1.932 | 0.0016 | 1.738 | 1.351–2.235 | **<0.0001** |
| Pathology of heart valves | 9371 | 2.106 | 1.458–3.044 | **0.0001** | 2.459 | 1.623–3.724 | **<0.0001** |
| Coronary heart disease, myocardial infarction, chronic heart failure | 9371 | 1.008 | 0.615–1.653 | 0.9735 | 1.243 | 0.805–1.919 | 0.3260 |
| Other | 9371 | 0.446 | 0.055–3.624 | 0.4497 | 0.810 | 0.202–3.240 | 0.7655 |
| Thyroid diseases | 9371 | 1.485 | 1.281–1.722 | **<0.0001** | 1.303 | 1.134–1.497 | 0.0002 |
| Hypothyroidism | 9371 | 1.609 | 1.346–1.924 | **<0.0001** | 1.488 | 1.250–1.771 | **<0.0001** |
| Nodular goiter | 9371 | 1.365 | 1.061–1.756 | 0.0155 | 1.143 | 0.905–1.444 | 0.2628 |
| Thyrotoxicosis | 9371 | 0.545 | 0.244–1.218 | 0.1389 | 0.713 | 0.401–1.266 | 0.2476 |
| Other | 9371 | 0.780 | 0.260–2.335 | 0.6568 | 1.214 | 0.496–2.972 | 0.6710 |
| Malignant neoplasms | 9371 | 1.915 | 1.162–3.156 | 0.0108 | 1.873 | 1.106–3.171 | 0.0195 |
| Stomach | 9371 | 1.041 | 0.108–10.008 | 0.9724 | 0.810 | 0.114–5.752 | 0.8330 |
| Lung | 9371 | n/a | n/a | n/a | 0.810 | 0.051–12.952 | 0.8815 |
| Other localization | 9371 | 1.471 | 0.634–3.412 | 0.3691 | 2.088 | 0.872–5.004 | 0.0986 |
| Other | 9371 | 2.056 | 1.151–3.673 | 0.0149 | 1.789 | 0.971–3.299 | 0.0623 |
| Other chronic diseases | 9371 | n/a | n/a | n/a | 1.336 | 0.969–1.842 | 0.0768 |
| **Obstetric and gynecological past medical history and status, Yes vs No** | | | | | | | |
| Diseases of the cervix and vulva | 9371 | 0.961 | 0.844–1.093 | 0.5431 | 1.192 | 1.066–1.333 | 0.0020 |
| Genital infection | 9371 | 1.095 | 0.933–1.285 | 0.2666 | 1.451 | 1.256–1.676 | **<0.0001** |
| Malformations of the genital organs | 9371 | 2.051 | 1.111–3.786 | 0.0217 | 2.101 | 1.078–4.095 | 0.0293 |
| Inflammatory diseases of the genital organs | 9371 | 1.040 | 0.903–1.197 | 0.5857 | 1.083 | 0.958–1.225 | 0.2020 |
| Dysmenorrhea | 9371 | 1.002 | 0.823–1.220 | 0.9819 | 1.119 | 0.944–1.328 | 0.1955 |
| Menstrual disorders |  |  |  |  |  |  |  |
| Amenorrhea, oligo-spaniomenorrhea | 9371 | 0.826 | 0.649–1.049 | 0.1174 | 1.065 | 0.874–1.298 | 0.5334 |
| Hyper-poly-menorrhea, abnormal uterine bleeding | 9371 | 1.240 | 1.034–1.487 | 0.0200 | 1.404 | 1.186–1.660 | **0.0001** |
| Premenstrual syndrome | 9371 | 1.048 | 0.904–1.214 | 0.5363 | 1.089 | 0.957–1.238 | 0.1954 |
| Regular menstruation | 9371 | 0.900 | 0.801–1.011 | 0.0762 | 1.059 | 0.959–1.169 | 0.2572 |
| Irregular menstruation | 9371 | 1.089 | 0.932–1.273 | 0.2835 | 1.201 | 1.046–1.379 | 0.0095 |
| Perimenopause | 9371 | 0.739 | 0.584–0.935 | 0.0116 | 0.949 | 0.787–1.144 | 0.5821 |
| Natural menopause | 9371 | 1.116 | 0.980–1.270 | 0.0975 | 1.086 | 0.969–1.217 | 0.1566 |
| Surgical menopause | 9371 | 1.289 | 1.069–1.555 | 0.0079 | 1.118 | 0.942–1.326 | 0.2036 |
| Climacteric syndrome | 9371 | 1.149 | 1.013–1.304 | 0.0313 | 1.268 | 1.132–1.419 | **<0.0001** |
| Prolapse of genital organs | 9371 | 1.282 | 1.036–1.587 | 0.0225 | 1.147 | 0.944–1.395 | 0.1684 |
| Urinary incontinence | 9371 | 1.671 | 1.311–2.129 | **<0.0001** | 1.453 | 1.145–1.844 | 0.0021 |
| Hyperplastic processes of the endometrium | 9371 | 1.371 | 1.133–1.659 | 0.0012 | 1.409 | 1.177–1.686 | 0.0002 |
| Endometriosis | 9371 | 1.284 | 1.130–1.459 | **0.0001** | 1.261 | 1.122–1.416 | **0.0001** |
| Uterine myoma | 9371 | 1.032 | 0.911–1.170 | 0.6193 | 1.120 | 1.004–1.248 | 0.0420 |
| Hyperprolactinemia | 9371 | 2.014 | 1.216–3.335 | 0.0065 | 1.355 | 0.815–2.251 | 0.2412 |
| Polycystic ovary syndrome | 9371 | 0.774 | 0.588–1.018 | 0.0665 | 1.036 | 0.830–1.293 | 0.7530 |
| Ovarian cysts | 9371 | 1.356 | 1.100–1.670 | 0.0043 | 1.351 | 1.111–1.644 | 0.0026 |
| Infertility | 9371 | 1.368 | 1.090–1.716 | 0.0068 | 1.606 | 1.290–1.999 | **<0.0001** |
| Complications of pregnancy and childbirth | 9371 | 1.325 | 1.172–1.498 | **<0.0001** | 1.457 | 1.301–1.631 | **<0.0001** |
| Ectopic pregnancy | 9371 | 1.177 | 0.839–1.652 | 0.3453 | 1.157 | 0.852–1.572 | 0.3508 |
| Syndrome of miscarriage of the fetus | 9371 | 1.893 | 1.414–2.535 | **<0.0001** | 1.677 | 1.243–2.263 | 0.0007 |
| Threatened miscarriage | 9371 | 1.205 | 1.040–1.397 | 0.0131 | 1.453 | 1.269–1.664 | **<0.0001** |
| Preeclampsia | 9371 | 2.325 | 1.739–3.108 | **<0.0001** | 3.047 | 2.152–4.314 | **<0.0001** |
| Feto-placental insufficiency | 9371 | 1.872 | 1.508–2.324 | **<0.0001** | 1.955 | 1.560–2.450 | **<0.0001** |
| Fetal death | 9371 | 1.110 | 0.626–1.968 | 0.7198 | 0.687 | 0.414–1.138 | 0.1446 |
| Premature birth | 9371 | 1.514 | 1.154–1.984 | 0.0027 | 1.330 | 1.025–1.725 | 0.0316 |
| Gynecological and obstetric operations | 9371 | 1.266 | 1.099–1.458 | 0.0011 | 1.317 | 1.157–1.499 | **<0.0001** |
| Diseases of the breast | 9371 | 0.745 | 0.474–1.173 | 0.2038 | 1.080 | 0.712–1.637 | 0.7177 |
| Diffuse form of fibrocystic mastopathy | 9371 | 1.253 | 1.102–1.426 | 0.0006 | 1.258 | 1.119–1.414 | **0.0001** |
| Nodular form of fibrocystic mastopathy | 9371 | 1.806 | 1.238–2.635 | 0.0022 | 1.320 | 0.909–1.919 | 0.1449 |
| Fibroadenoma | 9371 | 1.314 | 0.787–2.192 | 0.296 | 1.407 | 0.867–2.285 | 0.1668 |
| Cancer | 9371 | 1.812 | 0.861–3.814 | 0.1173 | 2.234 | 0.994–5.021 | 0.0518 |
| Malignant neoplasms of the genital organs | 9371 | 2.650 | 1.185–5.922 | 0.0176 | 3.090 | 1.153–8.283 | 0.0249 |
| Other gynecological diseases | 9371 | 0.953 | 0.702–1.294 | 0.7577 | 1.022 | 0.788–1.327 | 0.8683 |

N, number of patients included in the analysis.

^a^Estimated using logistic regression. ^b^Disregarding an allocation at the enrolment in the original studies. ^c^Including surgical menopause. ^d^Women of reproductive age with other hormonal conditions: endometriosis, polycystic ovarian disease, uterine leiomyoma, algodysmenorrhea, endometrial hyperplastic processes.

ALT, alanine aminotransferase; AST, aspartate aminotransferase; BMI, body mass index; CI, confidence interval; ECG, electrocardiogram; ENT, ear-nose-throat; FSH, follicle-stimulating hormone; HRT, hormone replacement therapy; LH, luteinizing hormone; OR, odds ratio.
